# Silicosis with bilateral spontaneous pneumothorax

**DOI:** 10.4103/0970-2113.68325

**Published:** 2010

**Authors:** Sanjay Fotedar, Dhruva Chaudhary, Vikas Singhla, Rajat Narang

**Affiliations:** *Department of Pulmonary Medicine and Critical Care, Pt. B.D. Sharma, Post Graduate Institute of Medical Sciences, Rohtak, India*

**Keywords:** Pneumothorax, reticulonodular shadows, silicosis

## Abstract

Presentation with simultaneous bilateral pneumothorax is uncommon and usually in the context of secondary spontaneous pneumothorax. The association of pneumothorax and silicosis is infrequent and most cases are unilateral. Bilateral pneumothorax in silicosis is very rare with just a few reports in medical literature.

## INTRODUCTION

Involvement of pleura in silicosis is rare and secondary spontaneous pneumothorax (SSP) is the only recognized pleural complication of silicosis. SSP occurs late in the course of disease and may prove a fatal complication along with underlying grossly compromised pulmonary function. SSP is uncommon with acute and accelerated silicosis.[1] The incidence of SSP in silicosis as such is not known. SSP is usually unilateral, but sometimes may present as bilateral pneumothorax which is rare presentation.[2,3] Authors report a case of bilateral SSP in a patient of accelerated silicosis.

## CASE REPORT

**Figure 1:**
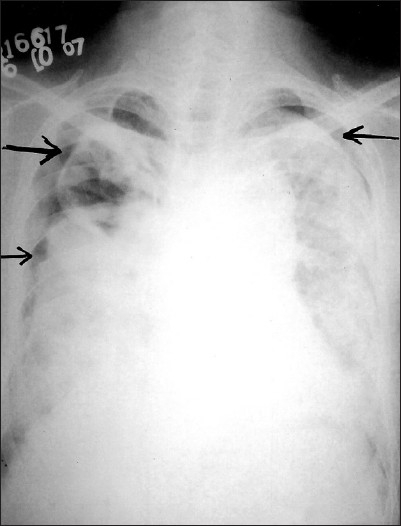
Chest radiograph (PA view) showing bilateral pneumothorax (arrows) with diffuse reticulonodular shadows)

**Figure 2:**
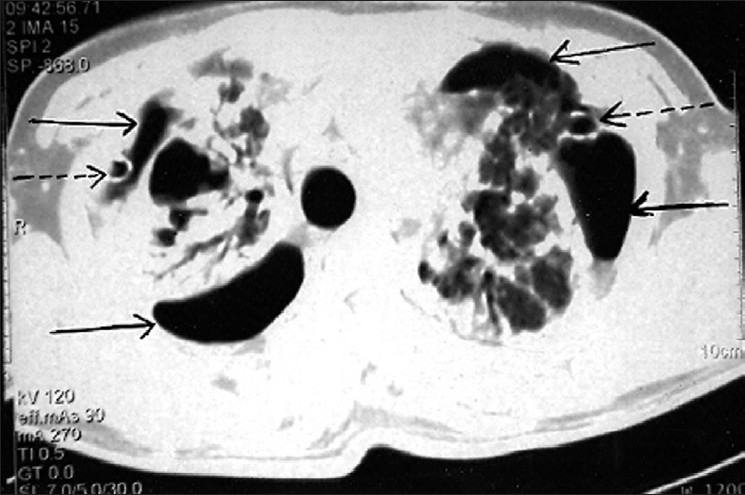
CT Thorax showing bilateral pneumothorax (solid arrows), bilateral chest tube in situ (interrupted arrows) along with reticulonodular shadows

**Figure 3:**
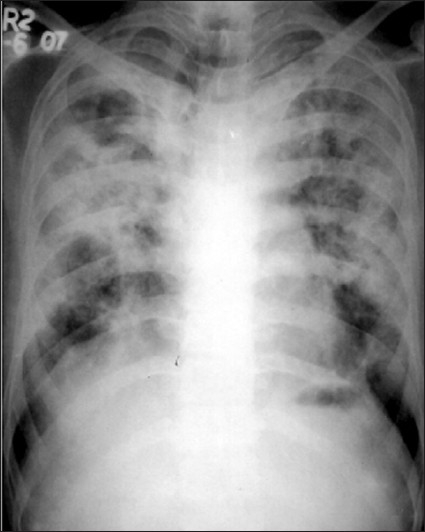
Chest radiograph (PA view) after chest tube removal showing fully expanded lungs with diffuse reticulonodular shadows

A 24-year-old male presented with acute breathlessness, bilateral chest pain and dry cough of two days duration. He complained of progressive exertional dysponea for last three years. Occupational history revealed that he had been working in the stone cutting industry for four years with an exposure to stone dust six hours a day. There was no history of haemoptysis, fever, skin rashes and joint pain. The patient was non-smoker. There was no history of contact with tuberculosis or antitubercular treatment. On examination, breath sounds were decreased in the upper chest areas with hyperresonant note and fine crepitations bilaterally. Laboratory findings were as follows Hb-12 gm%, total Leucocyte -1200 mm^3^, Polymorphs – 75 %, Lymphocytes – 24 %, Eosinophills – 1 %. Patient was non reactive for serum HIV. Chest radiograph showed bilateral diffuse reticulonodular shadows with bilateral pneumothorax [Figure 1]. As patient was in severe distress, active intervention was decided. Patient was managed by bilateral chest tube insertion under water seal and intermittent negative suction using pressure of 20–30 cm H_2_O; he improved clinically. Computerized tomography, planned to rule out any other underlying comorbid condition, revealed bilateral well defined nodular masses particularly in upper lung field with bilateral pneumothorax [Figure 2]. Sputum was negative for acid fast bacilli on three occasions and Mantoux test was negative. Ultrasonography thorax and abdomen was normal. Pleurodesis was done on both sides with 10% pivodine iodine and patient was discharged on day 15 after chest tube removal [Figure 3]. Follow-up remained uneventful for six months.

## DISCUSSION

The occurrence of occupational lung disease is decreasing due to improvement in occupational health in recent years, however, silicosis and its complications remain important occupational health problems.[2] Silicosis is a diffuse interstitial fibronodular involvement of lung caused by inhaling of crystalline silica. It results most commonly from exposure to quartz, enstobalite or tridymitesilica which is mainly associated with hard rock mining, silica mining, quarrying and stone work, foundry work, sand blasting, pottery making, glass making and cleaning boilers.[1]

Depending upon the intensity and duration of exposure, inhaled silica evokes three different types of reactions.[1] The first is chronic silicosis, the commonest form occurring after years of exposure to relatively low level of silica in the exposed environment. It is associated with progressive functional impairment with both restrictive and obstructive components and may include radiological features of progressive massive fibrosis. The second form, acute silicosis, occurs in heavily exposed environment and is quite severe with progressive course despite cessation of exposure. The clinical picture similar to alveolar proteinosis is an occasional development in these workers and is termed silicotic alveolar proteinosis. The third form, accelerated silicosis the intermediate type occurs after a few years of exposure and is characterized by rapidly progressive disabling form of disease.

Complications which occur in silicosis include lung infection, lung cancer, spontaneous pneumothorax and broncholithiasis. Crystalline silica, alpha quartz, less than 1µm in diameter is believed to be the most deadly pathogen.[4] Aerodynamic considerations appear to favor the entry and retention of particulates in the upper lobes of lungs. The particles are ingested by phagocytes, which accumulate and block lymphatic channels leading to the cascade of inflammatory process. The inflammatory process causes the weakening of elastica of alveolar walls leading to bleb formation and in some patients with congenital type II cell dysfunction to disease process indistinguishable from alveolar proteinosis, bleb formation may lead to pneumothorax.[3,5]

Secondary spontaneous pneumothorax (SSP) is a rare complication in the course of silicosis and usually unilateral.[2] SSP is seen in chronic silicosis having progressive massive fibrosis. It is rare event in acute silicosis. Functionally, massive fibrosis results in stiff nondistensable lungs with increased elastic recoil. Emphysema is common in patients with silicosis and is major cause of corpulmonale and disability. The development of SSP may be enhanced by increased elastic recoil of lungs parenchyma and bullae rupture.[2,6]

The predictive factors for development of SSP in patients with silicosis have not been extensively described in literature. In one study a strong association was found between the occurrence of SSP and presence of bullae. Silicosis is also associated with emphysematous changes in lungs, independent of smoking. In advanced silicosis coalescence of perinodular emphysematous regions may lead to formation of macroscopic blebs which rupture causing pneumothorax.[2]

Among the infections, tuberculosis is an important consideration in diagnosis and management of silicosis. It significantly contributes to the morbidity and mortality of patients with silicosis. The life time risk of tuberculosis is about 20–25% in patients of silicosis. Free silica impairs macrophage and microphage functions leading to increased chances of mycobacterial infections. It may also contribute to the development of progressive massive fibrosis in patients of silicosis. Because of strong association between silicosis and tuberculosis concurrent tubercular infection should be considered in patients with silicosis with relatively rapid clinical deterioration. The diagnosis of active tuberculosis in patient with silicosis demands high degree of clinical suspicion. Positive tuberculin test and cavitatory nodule are usually indicative of tuberculosis. CT scan of chest is also helpful in detection of concurrent tubercular infection. The treatment for concurrent tubercular infection in patients of silicosis requires prolonged duration.[5,7,8]

Nowadays, the diagnosis of silicosis is presumptive and based on combination of typical radiological features in chest radiograph, CT of thorax and occupational exposure followed by suitable latency period. The invasive procedures such as lung biopsy are taken only to exclude malignancy, rheumatoid nodules or infection.[5]

The cause of pneumothorax in our case was probably due to rupture of bleb, as there was no evidence of infection particularly tubercular. High degree of clinical suspicion in patients of silicosis when presenting with dysponea of acute onset should be investigated for this complication. The time of presentation and intervention may greatly reduce the morbidity and mortality and help patients lead purposeful and productive lives.
